# Editorial: Innovative robotics for lunar exploration and on-orbit servicing

**DOI:** 10.3389/frobt.2026.1835560

**Published:** 2026-04-09

**Authors:** Serdar Sean Kalaycioglu, Anton De Ruiter, Arun Misra, Kazuya Yoshida

**Affiliations:** 1 Department of Aerospace Engineering, Toronto Metropolitan University, Toronto, ON, Canada; 2 Mechanical Engineering, McGill University, Montreal, QC, Canada; 3 Tohoku University, Sendai, Japan

**Keywords:** autonomous robotics, lunar exploration, multi-robot systems, on-orbit servicing, robotic manipulators, space infrastructure, space robotics

The renewed international focus on lunar exploration and the rapid development of space infrastructure have created unprecedented demand for advanced robotic systems capable of operating reliably in extreme extraterrestrial environments. Over the past decade, initiatives such as NASA’s Artemis program and the planned Lunar Gateway station have highlighted the central role of robotics in enabling sustained human and robotic presence beyond Earth. Robotic technologies are essential for performing a wide range of tasks including planetary surface exploration, scientific data collection, autonomous navigation, infrastructure inspection, and on-orbit servicing of spacecraft and satellites.

The Research Topic “Innovative Robotics for Lunar Exploration and On-Orbit Servicing” was launched to highlight emerging research that advances robotic capabilities across both planetary surface missions and orbital space operations. Although lunar surface robotics and on-orbit servicing technologies are often studied independently, they are intrinsically linked within the broader architecture of future space exploration missions. Robotic systems operating on planetary surfaces enable terrain mapping, resource prospecting, and scientific investigation, while orbital robotic systems provide essential capabilities for spacecraft servicing, assembly, inspection, and maintenance of infrastructure that supports deep-space missions.

This Research Topic brings together four contributions that address key technological challenges in robotic autonomy, mission planning, exploration of extreme lunar environments, and robotic manipulation for space infrastructure maintenance.

One contribution investigates autonomous mission planning for planetary surface exploration using teams of micro-rovers. Swinton et al. propose a mission planning framework designed to coordinate cooperative teams of small robotic explorers operating in complex planetary environments. The proposed approach integrates terrain traversability analysis, probabilistic maps of scientific targets, and coordinated multi-agent path planning strategies. By employing algorithms such as RRT*-based path planning combined with prioritized coordination methods, the system enables multiple rovers to efficiently explore large regions while maintaining safe navigation constraints such as pitch and roll limitations. Distributed rover systems offer significant advantages over single large rover platforms, including improved mission resilience, redundancy, and expanded sensor coverage.

Another study addresses the exploration of lunar lava tubes, which are widely considered promising sites for future human habitation due to their natural shielding from radiation, micrometeorites, and extreme temperature variations on the lunar surface. Jagdish and Gunter present a feasibility study of a cold gas–propelled autonomous surveying vehicle designed to explore these subterranean environments. Unlike conventional wheeled rovers, the proposed vehicle utilizes a compact propulsion system based on low-pressure cold gas thrusters, enabling hovering and agile traversal across irregular and potentially hazardous terrain. The vehicle integrates onboard sensors such as LiDAR and inertial measurement units to support autonomous navigation and mapping of lava tube interiors. This concept illustrates how alternative robotic mobility systems may complement traditional rover technologies in exploring environments that are otherwise inaccessible or too hazardous for wheeled robotic platforms.

While planetary exploration robotics focuses primarily on mobility and autonomous operation on extraterrestrial surfaces, long-term space missions also require robotic technologies capable of supporting the maintenance and assembly of orbital infrastructure. Addressing this challenge, Quartaro et al. investigate the modeling and manipulation of deformable linear objects (DLOs), such as electrical cables, in robotic outfitting and maintenance operations. Flexible components present significant challenges for robotic manipulation because of their effectively infinite degrees of freedom and complex equilibrium configurations. The authors introduce a parametric multibody modeling framework combined with parameter estimation techniques derived from visual observations of cable configurations. Their approach balances computational efficiency with modeling accuracy by allowing adjustable discretization of the cable representation. This work contributes to the development of robotic systems capable of autonomously routing and manipulating flexible infrastructure components within complex space environments.

Complementing these focused technological advances, Alizadeh and Zhu provide a comprehensive survey of robotic manipulators for on-orbit servicing (OOS). Their review examines key technological areas necessary for successful robotic servicing missions, including object state estimation, motion planning, and feedback control strategies. The authors explore both classical robotics methodologies and emerging machine learning approaches that can enhance robotic perception and manipulation capabilities in microgravity environments. The survey also reviews the historical evolution of robotic manipulation technologies in space missions and highlights emerging applications such as satellite servicing, debris mitigation, refueling, and in-space assembly.

Taken together, the contributions in this Research Topic demonstrate the growing importance of robotics across the entire lifecycle of space missions—from initial planetary exploration and scientific investigation to the maintenance and servicing of orbital infrastructure. Advances in autonomy, sensing, modeling, and robotic manipulation are enabling robotic systems to operate more efficiently and reliably in the challenging conditions of extraterrestrial environments.

Looking forward, future research in space robotics will likely emphasize increasing levels of autonomy, improved robustness under harsh environmental conditions, and enhanced cooperation among heterogeneous robotic systems. Advances in artificial intelligence, perception technologies, and cooperative robotics are expected to further expand the capabilities of robotic platforms operating both on planetary surfaces and in orbit. Ultimately, robotic systems will serve as indispensable partners in humanity’s expansion into space. By bridging developments in lunar exploration robotics and on-orbit servicing technologies, the research presented in this Research Topic contributes to the broader goal of establishing sustainable and resilient space infrastructure to support long-term scientific discovery and exploration.

